# The science of elimination in practice

**DOI:** 10.1186/1475-2875-11-S1-O53

**Published:** 2012-10-15

**Authors:** Marcel Tanner

**Affiliations:** 1 Swiss Tropical and Public Health Institute, 4002 Basel, Switzerland; 2 University of Basel, 4003, Basel, Switzerland

## 

Malaria elimination and eradication is back on the global health agenda. The Roll Back Malaria *I* World Health Organization Global Malaria Action Plan sets out an ambitious plan for improved control, leading to regional elimination and includes as the ultimate goal the eradication of malaria. The ambitious goal of malaria eradication can however not be achieved with the tools and approaches currently available. Research and development of new tools must accompany and complement the Global Action Plan. We critically review and discuss the progress so far made to pursue the R&D agenda and about the key R&D issues where renewed attention and investments are required [1]. The following areas will be discussed on the basis of innovations made and practical applications already launched and validated.

– The strengthened focus on *P. vivax: in vitro* culture and study of the biology of hypnozoites.

– Drugs to be used for mass drug administration to clear infections and provide prophylaxis to prevent new infections Vaccines that aim at interrupting transmission.

– New vector control approaches for (i) outdoor biting / resting mosquitoes and (ii) achieving permanent reductions of vectorial capacity in areas where transmission is predominantly due to *A. gambiae.*

– New approaches for fast and accurate assessment of transmission at community level and strengthened diagnostic, monitoring, and surveillance tools/approaches that are linked and embedded in the health and social health systems.

– New approaches in mathematical modelling to inform Target Product Profiles of tools, and predict expected outcomes of intervention strategies for elimination.

– Tools to scientifically assess and determine health system readiness for moving from control to elimination.

The review will conclude by pinpointing the challenges that are still ahead of us in the development of the science for elimination and application of its results to effectively complement the Global Action Plan.
